# Supplementary material for: Mesenchymal Stem/Stromal Cells in Three-Dimensional Cell Culture: Ion Homeostasis and Ouabain-Induced Apoptosis
Source: Biomedicines. 2023 Jan 21;11(2):301. doi: 10.3390/biomedicines11020301 (PMC9953635; doi:10.3390/biomedicines11020301)
Supplement: Supplementary file 1 [file biomedicines-11-00301-s001.zip › biomedicines-2137611-supplementary.pdf]

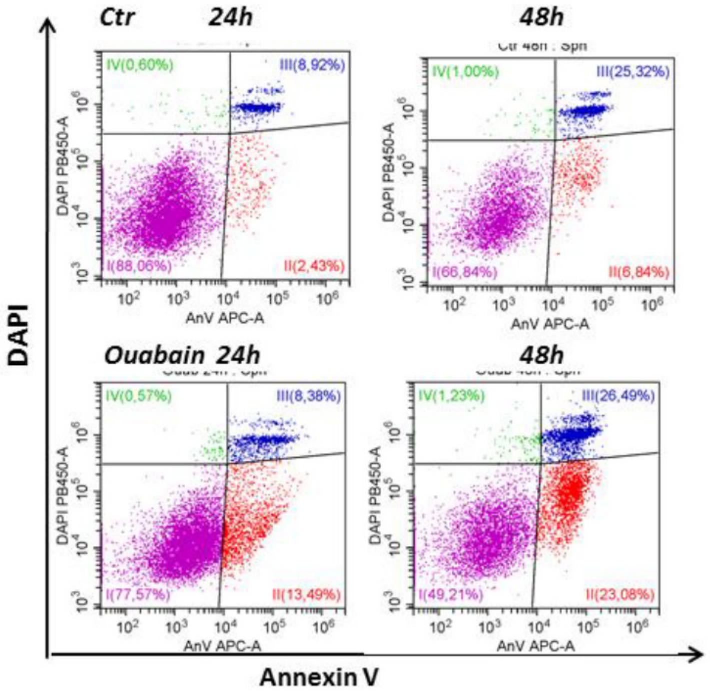

Figure S1. Apoptosis assay of eMSCs spheroids obtained using a hanging drop, thereafter transferred to dishes coated with 2-hydroxyethyl methacrylate (HEMA, Sigma) and cultured in 2 ml of complete growth medium with ouabain for the next 24 or 48 h. AnV-/DAPI- live cells (I); early apoptotic AnV+/DAPI- cells (II); late apoptotic cells AnV+/DAPI+ (III). DAPI+AnV- necrotic cells (IV).
